# A Pilot Study: Comparative Effects of Green Tea Extract and Duloxetine on Oxaliplatin-Induced Allodynia in a Murine Model

**DOI:** 10.3390/metabo15100680

**Published:** 2025-10-21

**Authors:** Michael Daniel, Stacie Totsch, Peng Li, Chisom O. Odii, Heather Shelton, Robert E. Sorge, Ellen Mary Lavoie Smith

**Affiliations:** 1 School of Nursing, University of Alabama at Birmingham, Birmingham, AL 35294, USA; 2 Department of Psychology, College of Arts and Sciences, University of Alabama at Birmingham, Birmingham, AL 35294, USA

**Keywords:** chemotherapy-induced peripheral neuropathy, CIPN, oxaliplatin, green tea, polyphenols, duloxetine, neuropathic pain, allodynia

## Abstract

**Background/Objectives:** The purpose of this preclinical pilot study was to explore the potential of green tea extract (GTE) to mitigate and/or prevent oxaliplatin-induced allodynia and axonal damage in rats, when compared to duloxetine (DLX), an ASCO-recommended treatment for established neuropathic pain. **Methods:** Using a randomized, placebo-controlled experimental design, Sprague Dawley rats (*N* = 41) received 4 intraperitoneal oxaliplatin (2 mg/kg) injections every other day over 7 days. One week prior to the first oxaliplatin dose, animals began 1 of 4 interventions (saline; GTE 100 mg/kg; DLX 3 mg/kg; GTE 100 mg/kg + DLX 3 mg/kg). A naïve group (*n* = 6) that received no neurotoxic oxaliplatin or intervention was added to serve as a baseline measure for sNfL. Interventions were administered daily for 4 weeks. Mechanical sensitivity (allodynia) was measured 3 times per week using von Frey testing to determine paw withdrawal thresholds. Von Frey testing began one day prior to the start of interventions to establish baselines and continued through Day 35. Groups were compared to their respective baselines to calculate changes in paw withdrawal thresholds. To measure axonal damage, alterations in serum levels of neurofilament light (sNfL) protein were measured at Day 35 using ELISA. Group differences were identified using two-way analysis of variance (ANOVA). Pearson correlation coefficient was used for correlation analysis between paw withdrawal thresholds and sNfL levels at Day 35. Partial eta-squared and Hedges’ g were used to measure effect sizes. Statistical significance was assigned at *P* ≤ 0.05 with a 95% confidence interval. **Results:** Overall, the saline group showed significant reductions in mean paw withdrawal thresholds across experimental timepoints, denoting more severe allodynia caused by oxaliplatin. Conversely, intervention groups exhibited mean paw withdrawal thresholds that were significantly greater than the saline group, indicating less allodynia. The average level of sNfL was also significantly higher in the saline group (113.58 ± 43.84 pg/mL) compared to GTE100 (72.75 ± 26.85), DLX3 (59.93 ± 20.57), and DLX3 + GTE100 (77.04 ± 24.35) intervention groups, suggesting less oxaliplatin-induced axonal damage in these groups. The naïve group exhibited the lowest levels of sNfL (45.69 ± 14.64) when compared to the oxaliplatin-receiving groups (saline and intervention). There were large effect sizes between the saline group, naïve (*g* = 1.88), GTE100 (*g* = 1.123), DLX3 (*g* = 1.157), and DLX3 + GTE100 (*g* = 1.030) groups. There was also a moderate negative correlation [*r*(30) = −0.38, *p* = 0.04] between sNfL levels and paw withdrawal thresholds. **Conclusions:** The preliminary findings from this pilot study suggest that GTE may be an effective, nutraceutical intervention for mitigating OIPN-associated neuropathic pain, warranting further investigation as an intervention to mitigate chemotherapy-associated neurotoxicities like OIPN.

## 1. Introduction

Chemotherapy is a mainstay cancer treatment received by a majority of cancer survivors living today. Chemotherapy-induced peripheral neuropathy (CIPN) is a prevalent and often debilitating side effect of neurotoxic chemotherapeutic agents (e.g., platinums, taxanes, vinca alkaloids) [1]. Oxaliplatin is a platinum-based, often neurotoxic, chemotherapeutic agent that is used in the management and treatment of colorectal cancer [2]. Although oxaliplatin has led to significant improvements in overall survival rates, more than 70% of patients develop sensory oxaliplatin-induced peripheral neuropathy (OIPN) [3,4,5]. Severe OIPN can pose a major health risk due to functional impairments, falls, depression, impaired sleep, and overall decreased quality of life. Due to the absence of effective preventative interventions, these patients often require chemotherapy dosage reductions to mitigate OIPN symptom severity, which can worsen survival outcomes [3,6,7,8].

Oxaliplatin damages peripheral sensory, motor, and autonomic nerves, with damage to the sensory nerves being most common [9]. OIPN is classified as either acute or chronic depending on the onset and persistence of symptoms, with acute OIPN occurring in 85–95% of patients treated with oxaliplatin. It often manifests as cold-induced symptoms like paresthesia (presence of numbness, tingling, and/or abnormal skin sensations) around the mouth, in the throat, and in the upper and lower extremities [10]. These symptoms can begin to manifest within hours of drug infusion and peak 3–5 days following chemotherapy cycle completion. Symptom severity increases as the cumulative chemotherapy dose increases and resolves before the next chemotherapy cycle begins [10,11]. On the other hand, chronic or long-term OIPN can persist for months to years after chemotherapy completion and even worsen in some instances in a phenomenon known as “coasting” [1]. Sensations of shooting and/or burning pain are described by some patients, with painful OIPN developing after numbness and tingling in approximately 30% of patients [12,13,14]. Painful OIPN is often characterized by either allodynia or hyperalgesia [14]. Allodynia is a neuropathic pain characteristic where pain is experienced due to stimuli that would not usually cause pain (e.g., light touch), whereas hyperalgesia describes exaggerated pain from stimuli that usually cause pain [15].

Although the exact pathophysiologic mechanisms surrounding the development of OIPN are still not fully understood, current thought is that mitochondrial damage and ion-channel dysfunction are underlying mechanisms [15,16,17]. Neuroaxonal damage is another proposed mechanism that may influence sensory OIPN in both acute and chronic states [18]. Neurofilament light chain (NfL) is a structural protein found in large, myelinated nerve axons [19]. Research suggests that neuroaxonal damage in peripheral nerves results in the release of NfL into extracellular space and peripheral blood. The levels of serum-present NfL (sNfL) can indicate initial and ongoing neuroaxonal injury caused by chemotherapy-induced neurotoxicity (CIN). Several studies have demonstrated that sNfL levels increase following neurotoxic chemotherapy treatment with vincristine in animals [20,21] and paclitaxel [22,23] and oxaliplatin [24] in patients with breast and colorectal cancer, respectively. Despite the variety of proposed pathophysiologic mechanisms, neuroaxonal degeneration appears to be the most convincing, final downstream pathway in the development of OIPN. Thus, understanding, measuring, and targeting chemotherapy-induced neuroaxonal degeneration, using sNfL as a measure, may be key to identifying effective interventions to prevent or mitigate OIPN.

Currently, duloxetine is the only American Society of Clinical Oncology (ASCO) recommended treatment for established neuropathic pain [25,26]. Duloxetine, a serotonin and norepinephrine dual reuptake inhibitor, has been shown to be effective in treating both diabetic and CIPN-associated neuropathic pain [8,26,27]. Neurotransmitters like serotonin and norepinephrine inhibit pain signal transmissions from spinal cord dorsal horn neurons to the brain [28,29,30]. Reuptake inhibition of these neurotransmitters by drugs like duloxetine increases the amount of both neurotransmitters in the pre/post-synaptic cleft and has been shown to be useful in treating/suppressing neuropathic pain [31]. In addition to inhibiting neurotransmitter reuptake, duloxetine acts locally by blocking Nav1.7 sodium channel currents [32,33]. Although duloxetine is recommended by ASCO for the treatment of established CIPN-associated neuropathic pain, it is underutilized and has not been shown to be effective in preventing CIPN in clinical settings [1,26,34]. As such, there remains a need for effective, alternative interventions to mitigate and/or prevent the often debilitating symptoms associated with CIPN, especially considering the emerging evidence in recent years regarding nonpharmacological interventions for the management and/or prevention of health conditions like neuropathic pain [35,36]. In addition, because treatment with antineoplastics like oxaliplatin is often a planned event, this allows preventive interventions to be employed proactively.

Phytocompounds are a promising class of bioactive, secondary metabolites widely found in plant sources that have gained recognition for their broad impact as modulators of human health and disease [37,38]. Polyphenols are a recognized class of phytocompounds, characterized by their phenolic structures, that can influence various physiological pathways. They have been shown to possess neuroprotective and neuroregenerative properties through their inhibition of oxidative stress and cellular damage, and modulation of ion channeling. Moreover, some metabolites have been shown to possess blood-brain barrier permeability, which could contribute to their neuroprotective effects [39,40]. Certain polyphenols can also influence the metabolism and action of various neurotransmitters and/or their receptors [41,42].

Tea (*Camellia sinensis*), a dietary source rich in polyphenols, is the second most consumed beverage in the world and has increasingly gained scientific recognition for its high concentration of a subclass of polyphenols known as catechins, which have been demonstrated to possess a variety of beneficial health properties [43]. Among these, epigallocatechin-3-gallate (EGCG) is the predominant green tea catechin, possessing various neuroprotective and neuroregenerative properties [40,44]. Several studies have demonstrated that EGCG provides protection against both central and peripheral nervous system injuries in preclinical models [45,46,47] and mitigates peripheral nerve damage in individuals with diabetic neuropathy [48]. The neuromodulating effects of green tea catechins (i.e., EGCG) are primarily attributed to their potent antioxidant properties, ability to regulate neurotrophic factors that are crucial for neurogenesis, and modulation of ion channel activities [49,50,51]. A known step in pathological neuroaxonal degeneration is the dysregulation of ion channels that leads to the buildup of ions and the subsequent breakdown of structural integrity and axonal degeneration [52]. Collectively, the literature on green tea and its catechin constituents, along with evidence from one other preclinical study [47], provide reasoned scientific support to study an intervention of GTE to target oxaliplatin-induced axonal damage and prevent sensory OIPN, for which there have been limited studies in this area.

Given the evidence of the neuroprotective and neuroregenerative properties of polyphenolic catechins like those abundantly found in green tea, further research is necessary to evaluate green tea extract (GTE) to mitigate OIPN-associated neuropathic pain. Thus, the primary aim of this study was to explore the potential of GTE to mitigate and/or prevent oxaliplatin-induced allodynia (as measured by paw withdrawal thresholds) and neuroaxonal damage (as measured by sNfL), when compared to a benchmark like duloxetine. For this pilot study, we chose a preclinical animal model of neuropathic pain for several reasons: (1) feasibility, (2) ability to test behavioral outcomes (e.g., paw withdrawal thresholds), and (3) translational value when compared to cellular models.

## 2. Materials and Methods

### 2.1. Animals

Experiments were performed on male and female Sprague-Dawley rats (8 weeks of age at onset; Charles River Labs, Wilmington, MA, USA) that were housed in groups of 3 rats/cage (10.25′′ W × 18.75′′ L × 8′′ H), in a 12:12 h light:dark cycle with lights ON at 0700 h. Standard chow (REG, NIH-31) and sterile water were provided. Animals were monitored daily throughout the experiment for general health, including daily weighing during oxaliplatin treatment and weekly thereafter. At the end of the protocol period, rats were humanely sacrificed. Animals that exhibited predefined hypersensitivity at baseline measurement were removed from the study and replaced with new cohorts. Animals were used in accordance with the University of Alabama at Birmingham Institutional Animal Care and Use Committee (IACUC) guidelines.

### 2.2. Experimental Design and Protocol

**Figure 1:**
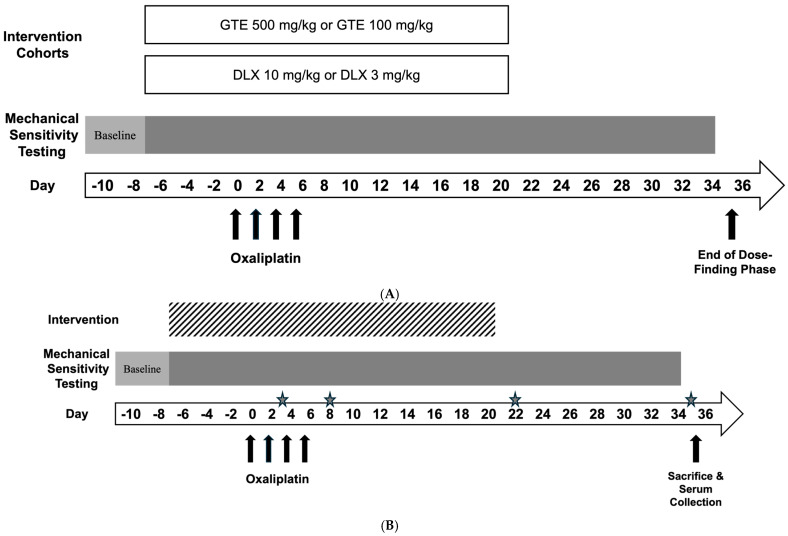
(**A**) Dose-finding Study Timeline. This schema represents the design of the dose-finding phase of the study. (**B**) Study Timeline. This is a representation of the experimental timeline, including intervention administration, oxaliplatin administration, mechanical sensitivity testing, and animal sacrifice. Stars indicate experimental time points where paw withdrawal thresholds were measured. The sNfL levels were measured at Day 35 only.

**Figure 2:**
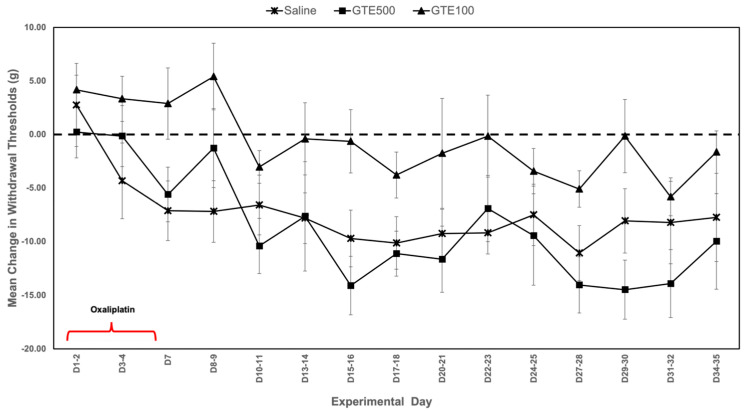
Dose-response component of the effects of GTE 500 mg/kg (GTE500) and GTE 100 mg/kg (GTE100) on paw withdrawal thresholds, as measured by Von Frey mechanical sensitivity testing and represented as mean change from baseline in paw withdrawal thresholds (in grams). Animals in the GTE100 group exhibited a mean change in paw withdrawal thresholds ± SEM that consistently suggested they were less allodynic than animals in both the saline and GTE500 groups.

An initial dose-response component (Figure 1A) was performed to determine optimal experimental doses where animals were randomized to either a GTE or duloxetine (DLX) cohort. In this phase, animals in each cohort were administered intervention, oxaliplatin, andunderwent mechanical sensitivity testing in accordance with study protocol. In the GTE cohort, rats were randomized to one of three arms: saline (*n* = 9), GTE 500 mg/kg (GTE500) (*n* = 5), or GTE 100 mg/kg (GTE100) (*n* = 10) (Figure 2). In the DLX cohort, rats were also randomized to one of three arms: saline (*n* = 9), DLX 10 mg/kg (DLX10) (*n* = 6), or DLX 3 mg/kg (DLX3) (*n* = 7) (Figure S1).

In the experimental component, a total of 41 Sprague-Dawley rats were randomized (via a random number generator) across five oxaliplatin-receiving cohorts: saline (*n* = 12), DLX3 (*n* = 7), GTE100 (*n* = 10), and a combination group: GTE100 + DLX3 (*n* = 6). Lastly, a naïve group (*n* = 6) was assigned to serve as a baseline/control group when measuring sNfL levels. Group sizes varied due to the listwise removal of outliers. Outliers were defined as those animals with paw withdrawal thresholds more than three standard deviations from the mean on more than 50% of the test days, as pre-specified in the protocol. Naïve animals received a standard chow diet, no oxaliplatin, and no experimental interventions. Baseline measurements were performed at the beginning of the experiment after the animals’ habituation period. Interventions were administered daily for 7 days prior to starting oxaliplatin injections. Interventions, in conjunction with oxaliplatin, were then administered for 7 days, after which oxaliplatin injections ceased and interventions continued for an additional 14 days post-oxaliplatin. The experiment ended on Day 35. For the purpose of data representation, 4 experimental timepoints were chosen to assess paw withdrawal thresholds (Figure 1B): Day 3, which was one day after the first oxaliplatin injection; Day 8 or approximately one day after the final oxaliplatin injection; Day 22, which was one day after stopping interventions and 2 weeks after the final oxaliplatin administration; and Day 35 (end of the experiment), which was two weeks after interventions ceased and four weeks post the final oxaliplatin injection.

### 2.3. Mechanical Sensitivity—Von Frey Filament Testing

Rats were placed in individual Plexiglas cubicles (custom-made, 4.25′′ W × 8.25′′ L × 4′′ H) atop a perforated metal floor. Animals were permitted to habituate to the testing environment for at least 30 min prior to testing. Nylon monofilaments (Stoelting Touch Test Sensory Evaluator Kit, fibers 2.0–60.0 g; Wood Dale, IL, USA) were applied to the plantar surface of the left or right hind paw for at least 1 s with a second-degree bend. Withdrawal thresholds were calculated using the up-down method [53]. Both hind paws were tested at least once on each test day and the values averaged to create a sensitivity score (mean paw withdrawal threshold, in grams). Changes in withdrawal threshold were calculated by subtracting animals’ timepoint thresholds from their respective baseline thresholds, and these values were averaged within groups to get mean changes in paw withdrawal thresholds. During the baseline period, a minimum of three measures per hind paw were taken to establish a stable baseline for comparison. Once administration of oxaliplatin and intervention(s) began, rats were tested 3 days/week until Day 35. Positive changes in paw withdrawal thresholds suggest no allodynia, as the threshold must have been higher than baseline. Withdrawal thresholds less than baseline suggest the development of and/or worsening of allodynia and are represented by negative changes in paw withdrawal thresholds.

### 2.4. Measurement of Serum Neurofilament Light

At the end of the protocol period (Day 35), rats were sacrificed, and cardiac blood was collected for sNfL measurement. Naïve rats were sacrificed to obtain a control/baseline sNfL measurement for comparison to oxaliplatin-treated animals. Whole blood was kept at room temperature for 30 min to allow clotting and then spun at 2000× *g* for 10 min. Serum was extracted and stored at −20 °C until needed for batch analysis. sNfL levels were quantitatively measured using UmanDiagnosistics (Umea, Sweden) NF-Light ELISA (Cat#: 20-8002). Samples were prepared and loaded into the UmanDiagnostics-supplied 96-well plate, in duplicate, and read using a microplate reader at an absorbance of 450 nm (reference wavelength 620–650 nm) in accordance with the manufacturer’s protocol.

### 2.5. Drug Administration

Oxaliplatin was purchased from Selleckchem (Houston, TX, USA) and dissolved in sterile saline to a final concentration of 1 mg/mL. Oxaliplatin solution was administered via intraperitoneal (IP) injection at a dose of 2 mg/kg every other day for a total of 4 injections and a total dose of 8 mg/kg, alternating body locations on each administration. A lower cumulative dose of oxaliplatin, when compared to other studies, was chosen in an effort to minimize systemic toxicity (e.g., weight loss) caused by oxaliplatin. This comparatively low dose of oxaliplatin was chosen based on previous reports of painful oxaliplatin-induced neuropathy in vivo models [47,54,55,56]. All rats, except those in the naïve group, received oxaliplatin treatment. Duloxetine hydrochloride was purchased from Sigma Aldrich (St. Louis, MO, USA) and diluted in sterile saline at a concentration of 15 mg/mL. Daily administration of duloxetine (3 mg/kg, IP; DLX3). Duloxetine 3 mg/kg is a human equivalent dose (HED) of approximately 30 mg of duloxetine for a 60 kg person [57], which was found to be therapeutic for painful chemotherapy-induced peripheral neuropathy [26]. Duloxetine administration began one week prior to oxaliplatin administration and continued for an additional 21 days (28 days total) (Figure 1). Body site was alternated on each day. Vehicle-treated animals received equivalent volumes of saline daily.

### 2.6. Diet Intervention Administration

Once stable baseline mechanical measures were obtained, rats randomized to the GTE intervention cohort were given a diet containing green tea extract (GTE, Sunphenon^®^ 90D, obtained from Taiyo International Inc., Minneapolis, MN, USA) at a concentration of 100 ppm (NIH-31 diet compounded with GTE by Inotiv, Madison, WI, USA, GTE100). Parts per million (ppm) dose translation was based on a previous dose translation report where 1 ppm represents 1 mg/kg of feed when diet is controlled [58]. Sunphenon 90D green tea powder is an extract of dried green tea leaves, *Camellia sinensis*, composed of highly purified polyphenols rich in natural green tea catechins. Sunphenon 90D is approximately 90% total polyphenols, more than 80% catechins (≥45% EGCG), and less than 1% caffeine [59]. A daily, controlled diet intervention of 100 mg/kg (100 ppm) GTE, which is a HED of approximately 972 mg GTE, has been previously shown to be both safe and tolerable in humans and animals [60,61,62,63]. This diet was given for one week prior to beginning oxaliplatin administration and continued for an additional 21 days (28 days total) until Day 21 (Figure 1).

### 2.7. Statistical Analysis

Animal cohorts consisted of between 6 and 12 rats. Data are presented as means ± SEM. Statistical comparisons between the intervention groups and across predefined experimental time points (Days 3, 8, 22, and 35) were made using two-way analysis of variance (ANOVA). Bonferroni test was used for post-hoc comparisons among means at specific timepoints. Pearson correlation coefficient was used for correlation analysis between paw withdrawal thresholds and sNfL levels at Day 35. Partial eta-squared or Hedges’ *g* was used to measure effect sizes. Significance was set at *P* ≤ 0.05, with a 95% confidence interval. The analyses were generated using IBM SPSS Statistics Version 29 (IBM Corp., Armonk, NY, USA) and graphs were prepared in Microsoft Excel (Version 2507; Redmond, WA, USA).

## 3. Results

### 3.1. Mechanical Sensitivity

In an initial dose-response component, preliminary doses of GTE (Figure 2) and duloxetine (Figure S1) were tested to determine optimal experimental doses, as decided by analyses of mean changes in paw withdrawal thresholds. During the first ten days of this component of the study, animals in both the GTE 100 mg/kg (GTE100) and GTE 500 mg/kg (GTE500) groups exhibited mean changes from baseline in paw withdrawal thresholds that were better than animals in the saline group. However, the allodynia-mitigating effects of GTE500 appeared to wane beginning Day 10–11, and this trend continued until Day 35. Conversely, animals in the GTE100 group consistently exhibited mean changes in paw withdrawal thresholds that were consistently better than those in both the GTE500 and saline groups. These findings suggested that GTE at a dose of 100 mg/kg is more effective at mitigating oxaliplatin-induced allodynia when compared to a higher dose of 500 mg/kg (Figure 2). A similar trend was also observed with duloxetine, where duloxetine 3 mg/kg (DLX) appeared to be more effective than duloxetine 10 mg/kg (DLX10) at mitigating oxaliplatin-induced allodynia (Figure S1). We observed no notable adverse side effects (e.g., weight loss) related to the interventions, which is consistent with reports from previous studies that used comparable yet higher doses of GTE [47] or duloxetine [34]. Following this dose-response component, we proceeded with the lower, seemingly more effective doses of GTE 100 mg/kg, duloxetine 3 mg/kg, and a combination of the two treatments for the remainder of the experiment’s protocol. A combination arm of the two most promising doses of GTE and DLX was done to preliminarily explore any competitive effects the interventions might exhibit related to the study’s outcome measures of paw withdrawal thresholds and chemotherapy-induced neuroaxonal damage as evidenced by sNfL.

**Figure 3:**
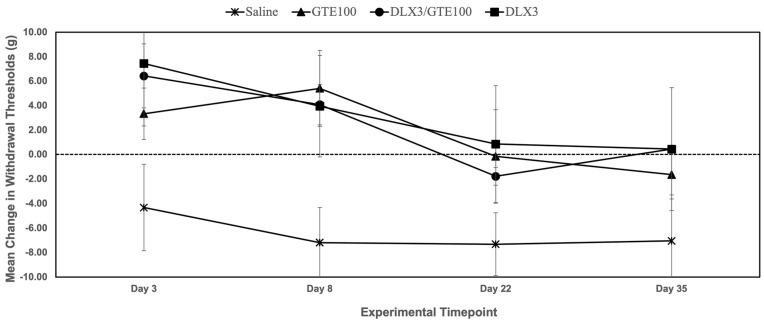
Effect of GTE 100 mg/kg, duloxetine (DLX) 3 mg/kg, and GTE 100 mg/kg + DLX 3 m/kg on paw withdrawal thresholds, as measured by Von Frey mechanical sensitivity testing and represented as mean change from baseline in paw withdrawal thresholds (in grams). Collectively, intervention groups were less allodynic, as evidenced by positive mean changes in paw withdrawal thresholds ± SEM when compared to animals in the saline group that showed negative mean changes in paw withdrawal thresholds.

Figure 3 and Table 1 show that animals who received intervention of GTE 100 mg/kg, DLX 3 mg/kg, or GTE 100 mg/kg + DLX 3 m/kg developed no mechanical hypersensitivity/allodynia until Day 22, as represented by positive mean changes in paw withdrawal thresholds. In contrast, saline-treated animals consistently exhibited negative changes in paw withdrawal thresholds (PWTs), translating to worsening hypersensitivity across experimental timepoints. Within-group comparison of mean changes in paw withdrawal thresholds among experiment groups revealed a statistically significant difference [*F* (5,149) = 5.629, *p* = < 0.001]. A large effect size was observed within groups (η^2^ = 0.159), and a medium effect was observed with experiment timepoints (η^2^ = 0.096), However, a statistically significant interaction between experiment timepoint and intervention group was not demonstrated [*F* (15,149) = 0.46, *p* = 0.96], but a small-to-medium effect size was observed (η^2^ = 0.044). Bonferroni post-hoc analysis revealed significant differences between timepoints Day 3 and Day 35 (*p* = 0.011).

Unlike animals in the intervention groups, allodynia continued to worsen in the saline group, which is consistent with an oxaliplatin-induced neurotoxicity phenomenon known as “coasting” seen in humans [1]. Pretreatment, prior to oxaliplatin administration, with intervention appeared to offer some protective effect against the development of severe allodynia. No statistically significant sex differences were observed among groups, and the sexes were collapsed.

### 3.2. Serum Neurofilament Light

**Figure 4:**
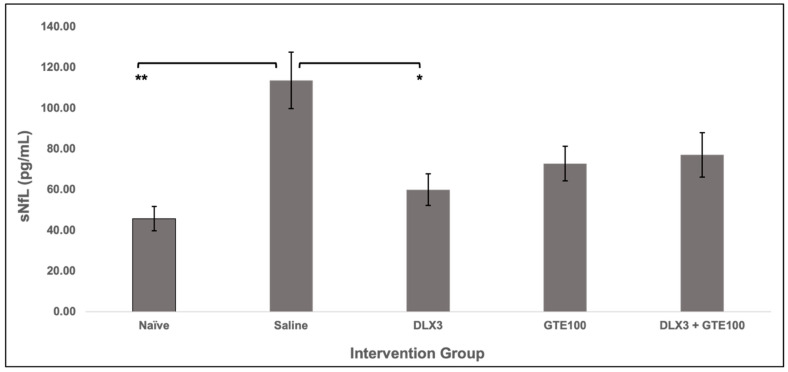
Average sNfL levels at Day 35 (end of experiment). Animals in the saline group had significantly higher levels of sNfL when compared to those in the naïve and intervention groups. Significance (**) *p* ≤ 0.01; (*) *p* ≤ 0.05; 95% confidence interval.

Figure 4 represents the average sNfL levels among oxaliplatin-received intervention animals and oxaliplatin-naïve animals. At Day 35, significant differences between groups were observed (*F*(4,33) = [5.74], *p* = 0.01). Naïve animals had the lowest mean sNfL level (45.69 ± 5.97 pg/mL). Animals in the saline group had the highest mean sNfL level (113.58 ± 13.86 pg/mL), which was significantly greater than all other groups, denoting the most oxaliplatin-induced axonal damage among these animals. The DLX3 group had an average sNfL level of 59.93 ± 7.78 pg/mL, the GTE100 group 72.75 ± 8.49 pg/mL, and the DLX3 + GTE100 group 77.04 ± 10.89 pg/mL. Within-group comparisons revealed a statistically significant difference [*F*(4,33) = 5.95, *p* = 0.001]. Tukey’s HSD post hoc analysis showed a statistically significant difference between the naïve and saline group (*p* = 0.001), as well as the saline and duloxetine group (*p* = 0.016). Large effect sizes were also observed among the groups: saline compared to naïve (*g* = 1.188), GTE100 (*g* = 1.123), DLX3 (*g* = 1.567), and DLX3 + GTE100 (*g* = 1.030). These data suggest less axonal damage in the intervention groups when compared to the saline group. No significant difference in mean sNfL levels was observed between naïve and intervention groups. No statistically significant sex differences were observed among groups, and the sexes were collapsed. A moderate negative correlation (*r*(30) = −0.38, *p* = 0.04) between sNfL levels and paw withdrawal thresholds was observed.

## 4. Discussion

Despite its pervasiveness among cancer patients and survivors, there are no known interventions that prevent painful OIPN [1,26]. Dose reductions remain the primary clinical approach to alleviate OIPN-associated sensory symptoms like neuropathic pain, with duloxetine being the only recommended treatment for established, painful OIPN [1,26]. Although duloxetine as an effective and evidenced treatment for existing neuropathic pain, the associated adverse events, perceptions and attitudes towards antidepressant/antianxiety medications like duloxetine, and the multifactorial stigma surrounding their use must be acknowledged [64,65]. Broadly, these factors and others may limit its use and incorporation into standard clinical practices for the treatment of chemotherapy-associated neuropathic pain. As such, there remains a need for effective, alternative, treatments or interventions for the management of OIPN-associated sensory symptoms like neuropathic pain [64,66]. Thus, this study sought to first explore the potential of GTE to mitigate and/or prevent oxaliplatin-induced allodynia, as measured by paw withdrawal thresholds and a blood biomarker of axonal damage, sNfL, in a rat model, and second to compare the effects of GTE to those of duloxetine, the benchmark treatment for existing neuropathic pain.

The results of this pilot study show that GTE can mitigate chemotherapy-induced allodynia in animals administered neurotoxic levels of oxaliplatin. Animals in the GTE 100 mg/kg intervention group exhibited no mechanical hypersensitivity/allodynia while receiving intervention during the oxaliplatin administration phase. This preventative property was also observed in the DLX 3 mg/kg and combination intervention groups. This suggests that an intervention of GTE 100 mg/kg is as effective as DLX 3 mg/kg at mitigating oxaliplatin-induced allodynia in this model. This observation may be attributed to, at least in part, the one week of pretreatment with intervention before first oxaliplatin administration, which may have extended some additional protective effect. Mechanistically, the catechins in GTE (i.e., EGCG) can target and/or interact with various ion channels that are similarly influenced by duloxetine. Previous studies have demonstrated the effects of EGCG on both calcium and sodium channel signaling in neuronal cells [67,68]. Similarly, duloxetine has been demonstrated to block neuronal sodium ion channels [69,70]. This potential sharing of mechanistic targets or pathways offered a rationale to explore the effects of a combination of GTE and duloxetine. The goal of this intervention arm was not to directly examine any additive or synergistic effects of a combination but instead to gather initial evidence of no competitive effects, as this may have larger clinical implications. Despite the diversity of additional mechanisms that may lead to the development of OIPN, there is convincing evidence that a final, common downstream pathway in the development of OIPN is neuroaxonal degradation [18,71]. Understanding, measuring, and targeting neuroaxonal degradation will be key to identifying effective interventions for OIPN.

The incorporation of objective measures when exploring neuropathic pain in animal models is essential due to the variability in behavioral sensory measurements like paw withdrawal thresholds that are commonly used to capture specific sensory abnormalities like mechanical hypersensitivity or allodynia [72]. Accordingly, exploration of changes in serum levels of NfL was done to quantify oxaliplatin-induced axonal damage that influences the development and severity of allodynia. Although sNfL is a single, terminal measurement of axonal damage and there could be other markers of neuroaxonal damage or degradation, there is consensus that sNfL is the most promising biomarker to indicate and quantify chemotherapy-induced neuroaxonal damage in both preclinical and clinical studies [73,74]. It was observed in this pilot study that oxaliplatin causes neuroaxonal damage, as evidenced by the spike in sNfL when compared to oxaliplatin-naïve animals. It has been suggested that oxalate, a metabolite of oxaliplatin, chelates calcium, making it unavailable for ion channel binding, which leads to calcium-dependent sodium channel dysfunction and a buildup of calcium ions. This buildup then leads to the activation of calpains, which rapidly proteolyze axonal cytoskeletons and causes axonal degradation [75,76,77].

The observation of sNfL levels being lower in the intervention groups when compared to the saline group, as well as intervention groups exhibiting lower changes from baseline in paw withdrawal thresholds, may be explained by the known ion channel-modulating properties of both GTE and duloxetine. This convergence of ion-modulating properties may also explain the absence of observable additive effects in the combination intervention arm. This concept of saturation related to an intervention’s limitation to further modulate ion channeling may also explain why higher concentrations of GTE and duloxetine did not result in better paw withdrawal thresholds in the dose-finding component. The fact that intervention animals were pretreated with a week of intervention prior to oxaliplatin introduction could have added to this hypothesized saturation phenomenon related to the presence and severity of allodynia. This pretreatment might also explain why no obvious additive effects were observed in the combination intervention group. Given the pharmacokinetic profile of green tea catechins related to bioavailability and short half-life in tissues and cells [78], pretreatment may have aided in bolstering the effects of our comparatively conservative intervention dosages through an existing blood or tissue concentration of GTE or duloxetine prior to oxaliplatin. These hypotheses may also lend some explanation to why lower concentrations of GTE and duloxetine were more effective at mitigating oxaliplatin-induced allodynia when compared to higher concentrations. Biologically relevant is the observation of no statistically significant differences in sNfL levels between naïve and intervention groups. This suggests that GTE and duloxetine mitigate oxaliplatin-induced neuroaxonal damage to levels that are statistically indistinguishable from homeostatic levels. However, this pilot study is neither adequately designed nor powered to conclude this and additional studies would need to be performed for confirmation. These findings provide preliminary empirical support regarding the promise of nutraceutical interventions like GTE to mitigate or prevent OIPN sensory symptoms like neuropathic pain. Moreover, the use of a biomarker of neuroaxonal damage, which is a hypothesized, final downstream mechanism of chemotherapy-induced neuropathic pain, to measure intervention response will be useful in future studies, potentially mitigating the risks of placebo effects in clinical settings while also increasing the integrity and validity of the research.

Despite the promising results of this study, there were a few limitations. First, this study was performed in nontumor-bearing animals. However, it should be noted that preclinical murine models of neuropathic pain are well-established. Moreover, sNfL as a biomarker of oxaliplatin-induced neuroaxonal damage has been demonstrated in both preclinical models as well as cancer patient populations. Exploration into any antagonistic properties of GTE and the efficacy of oxaliplatin as an antineoplastic was not conducted. However, previous studies have demonstrated that the combination of chemotherapeutic drugs (e.g., cisplatin and paclitaxel) and green tea extract or tea polyphenols can synergistically enhance treatment efficacy [79], as well as reduce the adverse side effects of antineoplastic drugs in patients with cancer [80,81,82,83]. The primary outcome was the prevention and/or mitigation of oxaliplatin-induced neuropathic pain, as measured by the presence of OIPN-associated mechanical hypersensitivity (allodynia). However, measuring only allodynia and not also hyperalgesia limited the study to a singular sensory behavioral measure. We sought to assuage this by incorporating an objective sensory profile biomarker of axonal damage, sNfL. A limitation of this approach was that sNfL was only measurable at one timepoint (Day 35, end of the experiment), due to the volume of whole blood required to obtain adequate volumes of serum for analysis. It would have been ideal to measure sNfL at multiple timepoints to explore associations between paw withdrawal thresholds and sNfL levels across experimental timepoints.

## 5. Conclusions

The results from this pilot study provide preliminary data that demonstrates an intervention of oral GTE may be an effective mitigator of oxaliplatin-induced allodynia and a potential nutraceutical alternative to duloxetine for OIPN-associated sensory symptoms like neuropathic pain and warrants further, robust exploration in varying models of chemotherapy-associated neurotoxicities and neuropathic pain. Also, the incorporation of biomarker measures, like sNfL, in future CIPN intervention studies may aid in strengthening the validity and rigor of future clinical research, which is often plagued by measurement variability.

## Figures and Tables

**Table 1:** Mean changes in paw withdrawal thresholds as measured by Von Frey Mechanical Sensitivity Testing.

Group	Day 3	Day 8	Day 22	Day 35
GTE 100 mg/kg	+3.33 ± 2.11 g (*NS*)	+5.41 ± 3.11 g (*p =* 0.01) *	−1.78 ± 0.73 g (*NS*)	−1.64 ± 1.98 g (*NS*)
DLX 3 mg/kg	+7.44 ± 5.10 g (*NS*)	+3.95 ± 4.15 g (*p =* 0.02) *	+0.86 ± 4.77 g (*NS*)	+0.45 ± 5.02 g (*NS*)
GTE 100 mg/kg + DLX 3 mg/kg	+6.43 ± 2.62 g (*NS*)	+4.08 ± 1.66 g (*p =* 0.01) *	−1.78 ± 0.73 g (*NS*)	+0.45 ± 0.20 g (*NS*)
Saline	−4.33 ± 3.53 g	−7.19 ± 2.87 g	−7.32 ± 2.56 g	−7.04 ± 3.75 g

* Statistical significance is in comparison to Saline at respective experimental timepoints; (*NS*) = no significance; (g) = grams.

## Data Availability

Dataset available on request from the authors.

## Supplementary Materials

The following supporting information can be downloaded at: https://www.mdpi.com/article/10.3390/metabo15100680/s1, Figure S1: Dose-response component of the effects of DLX 10 mg/kg and DX 3 mg/kg on paw withdrawal thresholds, as measured by Von Frey mechanical sensitivity testing and represented as mean change from baseline in paw withdrawal thresholds (in grams).
