# Supplementary material for: A Pilot Study: Comparative Effects of Green Tea Extract and Duloxetine on Oxaliplatin-Induced Allodynia in a Murine Model
Source: Metabolites. 2025 Oct 21;15(10):680. doi: 10.3390/metabo15100680 (PMC12566373; doi:10.3390/metabo15100680)
Supplement: Supplementary file 1 [file metabolites-15-00680-s001.zip › metabolites-3848088-supplementary.pdf]

# Supplementary Materials

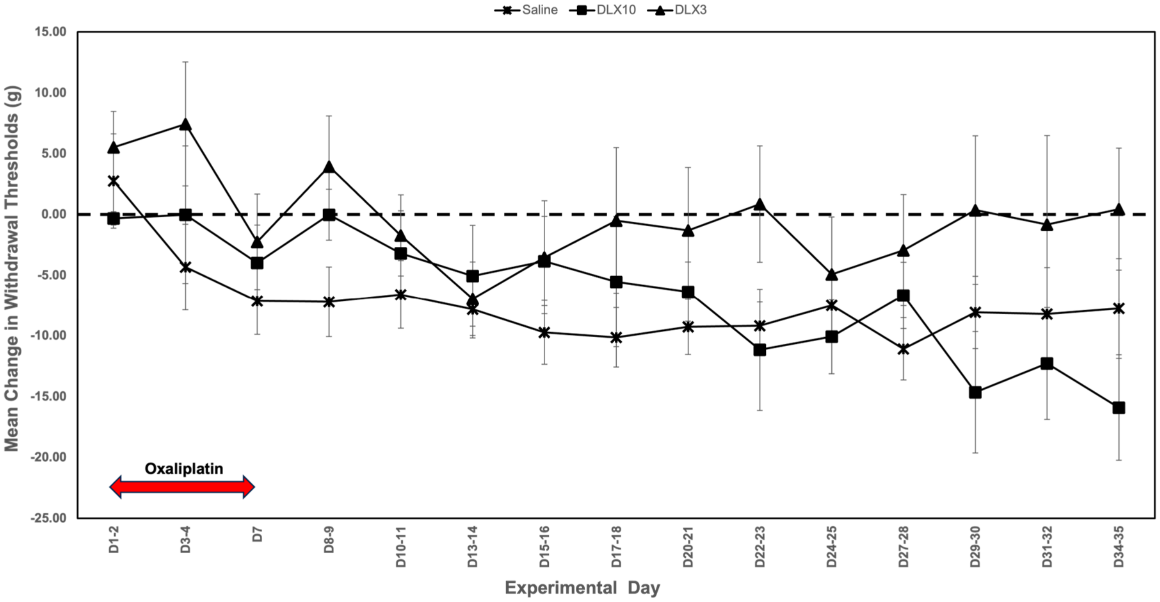


**Figure S1.** Dose–response component of the effects of DLX 10 mg/kg and DX 3 mg/kg on paw withdrawal thresholds, as measured by Von Frey mechanical sensitivity testing and represented as mean change from baseline in paw withdrawal thresholds (in grams).
